# The Role of IFN-β during the Course of Sepsis Progression and Its Therapeutic Potential

**DOI:** 10.3389/fimmu.2017.00493

**Published:** 2017-05-08

**Authors:** Gorjana Rackov, Rahman Shokri, Melchor Álvarez De Mon, Carlos Martínez-A., Dimitrios Balomenos

**Affiliations:** ^1^Department of Immunology and Oncology, Universidad Autónoma de Madrid, Centro Nacional de Biotecnología – CSIC, Madrid, Spain; ^2^IMDEA Nanoscience, Universidad Autónoma de Madrid, Madrid, Spain; ^3^Immune System Diseases-Rheumatology and Oncology Service, University Hospital Principe de Asturias, Alcalá de Henares, Spain

**Keywords:** IFN, IFN-β, sepsis, macrophages, monocytes, immunosuppression, M1–M2 polarization, p21

## Abstract

Sepsis is a complex biphasic syndrome characterized by both pro- and anti-inflammatory immune states. Whereas early sepsis mortality is caused by an acute, deleterious pro-inflammatory response, the second sepsis phase is governed by acute immunosuppression, which predisposes patients to long-term risk for life-threatening secondary infections. Despite extensive basic research and clinical trials, there is to date no specific therapy for sepsis, and mortality rates are on the rise. Although IFN-β is one of the most-studied cytokines, its diverse effects are not fully understood. Depending on the disease or type of infection, it can have beneficial or detrimental effects. As IFN-β has been used successfully to treat diverse diseases, emphasis has been placed on understanding the role of IFN-β in sepsis. Analyses of mouse models of septic shock attribute a pro-inflammatory role to IFN-β in sepsis development. As anti-inflammatory treatments in humans with antibodies to TNF-α or IL1-β resulted disappointing, cytokine modulation approaches were discouraged and neutralization of IFN-β has not been pursued for sepsis treatment. In the case of patients with delayed sepsis and immunosuppression, there is a debate as to whether the use of specific cytokines would restore the deactivated immune response. Recent reports show an association of low IFN-β levels with the hyporesponsive state of monocytes from sepsis patients and after endotoxin tolerance induction. These data, discussed here, project a role for IFN-β in restoring monocyte function and reversing immunosuppression, and suggest IFN-β-based additive immunomodulatory therapy. The dichotomy in putative therapeutic approaches, involving reduction or an increase in IFN-β levels, mirrors the contrasting nature of the early hyperinflammatory state and the delayed immunosuppression phase.

## Introduction

Sepsis is a systemic inflammatory syndrome caused by massive microbial infections and is a major cause of death worldwide. Sepsis is defined as an “organ dysfunction caused by a dysregulated host response to infection,” while septic shock is associated to a greater mortality risk, caused by “underlying circulatory, cellular and metabolic abnormalities” (1, 2). Although survival of sepsis patients with overwhelming pro-inflammatory responses is greatly improved in intensive care units (ICU), most patients develop delayed sepsis with severely suppressed immune responses and succumb to secondary infections (3, 4).

IFN-β is an essential cytokine in promoting and regulating innate and adaptive immune responses; its potential as an antimicrobial agent has been studied extensively. Data from murine models have assigned IFN-β a role in septic shock development, and its neutralization is proposed as a therapeutic strategy for human sepsis (5). As recent findings show low IFN-β expression by non-responsive monocytes in delayed sepsis patients, we discuss the therapeutic value of blocking or enhancing the levels of this cytokine to modulate immunosuppression.

### Sepsis Progression from Hyperinflammation and Early Death to Immunosuppression and Delayed Death

Following massive microbial infection, highly produced inflammatory cytokines, mainly TNF-α and IL-1β, drive hyperinflammation in sepsis patients (6, 7). Patients can suffer early death several days after systemic infection, due to sepsis and septic shock (8). Improvement in ICU care and compliance with the “Surviving Sepsis Campaign,” which provides clinical practice guidelines for the recognition and management of sepsis and septic shock, has reduced death incidence of sepsis patients (9–11). Early sepsis patients that survive ICU can nonetheless develop delayed sepsis and immunosuppression (12, 13). Patient death can be prolonged after initial sepsis diagnosis, due to a deactivated immune response (14, 15).

Hyporesponsiveness is considered a counteracting mechanism that regulates hyperinflammation and alleviates the deleterious effects of primary infection (16, 17). This state correlates with sepsis progression and death, as it is associated with increased risk for secondary nosocomial infections (3). In a recent review, Delano and Ward (18) present the evolution of mortality as early and late deaths and introduce a third modality of sepsis, long-term death, which can be delayed for years. As the incidence of early deaths in the ICU has diminished over the years (4), the burden of sepsis-related deaths is linked to the hyporesponsive phase of the syndrome, and late and long-term deaths are on the rise (19). The progression from initial sepsis to the prolonged syndrome is not clearly defined and the host response to sepsis might consist of concurrent inflammatory and immunosuppression processes (8, 20). Patients were recently identified that develop persistent inflammation-immunosuppression and catabolism syndrome (PICS), which leads to ongoing organ injury and death (13, 18, 21).

### Mouse Models for Analysis of the Biphasic Aspects of Sepsis

Mouse models are valuable tools with which to dissect the mechanisms of human disease, and aid in discovering innovative therapeutic advances. In sepsis, there is nonetheless a disparity at the molecular level between mouse models and the human syndrome (22–25), and human and mouse inflammatory diseases show low gene correlation (26). Findings from these models must thus be evaluated critically for applicability to human sepsis.

Injection of lipopolysaccharide (LPS, also termed endotoxin), a constituent of Gram-negative bacteria, leads to septic shock in mice. Infection models or cecal ligation and puncture (CLP) (27) also lead to septic shock. Compared to the widely used LPS model, the CLP model is a more clear approximation of polymicrobial sepsis as it leads to bacteremia, a feature shared with human sepsis (27).

Mouse systems that emulate hyporesponsiveness and delayed sepsis in humans are limited due to the inherent complexity of sepsis and to its heterogeneity. Non-lethal CLP models adapted to restrict death of treated mice can also be used to study delayed sepsis [see review by Dejager et al. (27)]. Mice subjected to mild CLP induction or CLP models treated with antibiotics survive early hyperinflammation and show long-term immune dysfunction. Exposure of these mice to secondary bacterial infection establishes the “two-hit” model that allows the study of compromised responses (28).

Endotoxin tolerance is a convenient model for analysis of macrophage hyporesponsiveness; it is induced after exposure of mice to a non-lethal LPS dose that induces hyperinflammation (29, 30). In a few hours, macrophages from treated mice undergo functional reprogramming from activated M1 status to an M2 hyporesponsive phenotype, and epigenetic modifications could explain this polarization (31–34). Endotoxin-tolerant macrophages are hyporesponsive to subsequent LPS challenge, and produce low amounts of TNF-α, IFN-β, and inducible nitric oxide synthase (iNOS) (34). This system deviates from the CLP model and from basic human sepsis features, as it is limited to the effects of LPS and not bacterial infection, and the initial LPS treatment does not lead to trauma and death.

As *in vitro* LPS stimulation induces endotoxin tolerance in human monocytes (31, 35) and their refractory state shows a certain analogy to monocyte hyporesponsiveness in sepsis patients (6, 15, 20), data from this model may be useful, but are considered preliminary (16, 24) and should be verified in CPL models and in human sepsis.

## Treatment Strategies for Sepsis: Past and Present

To date, intense research in the field has provided effective approaches for early sepsis treatment that have increased survival in the ICU (3, 21); there have nonetheless been no therapeutic advances for long-term sepsis-related immunosuppression.

To minimize the pro-inflammatory condition of sepsis patients, it seemed logical to antagonize hyperinflammation and to treat sepsis by neutralizing hyperinflammation through anti-TNF-α or -IL-1β specific antibodies (7). In mice, anti-TNF-α delivery protected from septic shock when delivered before or simultaneously with LPS, although patients treated with anti-TNF-α or -IL-1β antibodies failed to show sepsis improvement (7, 36). Perhaps, therapeutics directed to the early physio-pathological conditions that derive from this initial pro-inflammatory response would be more efficient in preventing early death in sepsis patients. One such condition is the activation of procoagulant pathways (27).

The ideal treatment would be based on an approach that could remedy both phases of sepsis (37). Because of the contrasting nature of early and delayed sepsis, distinct therapeutic approaches are currently considered to control hyperinflammation or immunosuppression. Before treatment, the state of each patient should thus be taken into strict account and tested, for example, by measuring HLA-DR expression in myeloid cells and evaluating overall immune cell status (3, 18).

As early sepsis survivors eventually develop immunosuppression, there is particular interest in establishing interventions for the late sepsis phase and a debate as to whether treatment for such patients should focus on boosting the pro-inflammatory response (7, 37). Macrophages are directly associated with sepsis development since Gram-negative bacteria, major constituents of infection, promote their activation through the TLR4 receptor; macrophages can then acquire a deactivated status (16). Other immune components participate in immunosuppression development in delayed sepsis. For example, T cell numbers decline due to apoptosis and attain an exhaustion state or impaired function, whereas T regulatory cells (Treg) are associated with mortality of delayed sepsis patients. NK cells and neutrophils have altered signaling functions. Moreover, a population of immature myeloid-derived suppressor cells (MDSC) arises and promotes immunosuppression (8, 18, 21). Dendritic cells undergo apoptosis in sepsis (38–41) and subsequently reemerge, but their activity is compromised due to the production of anti-inflammatory IL-10 (42–45). Because of these diverse immunosuppression features, it was suggested that intervention should not be limited to targeting a single affected immune component, but rather implement combination approaches to improve critical immune defects of sepsis-affected individuals (18). Such methods would imply delivery of immune modulators such as G-CSF, GM-CSF, IFN-γ, or IL-15 (7, 18), to reconstitute specific immune deficiencies that depend on sepsis stage and the patient’s needs.

## The Role of IFN-β in Immune Disease and in Sepsis

### IFN-β in Infection and Disease: Beneficial and Harmful Effects

The interferons are cytokines that modulate the immune response and antimicrobial infection; they are classified as types I, II, and III. IFN-γ is the only type-II cytokine, and IFN-α and -β (IFNα/β) of the broad IFN I family are the most studied. After microbial infection, endothelial, epithelial, and immune cells detect through their pattern-recognition receptors (PRRs), which include TLR, pathogen-associated molecular patterns (PAMPs). This interaction promotes IFN-β, which is produced by most nucleated cells (46, 47). All type-I IFNs bind to the same cell surface IFN-α/β receptor (IFNAR). Signaling through IFNAR initiates a cascade of events, which activates innate cells, and elicits chemokine/cytokine production and activation of adaptive immunity (47). IFNα/β was initially found to have antiviral activity, as their defective signaling increases viral susceptibility (48–53). Accordingly, IFN-α is effective for treatment of viral infections such as hepatitis C (54), and IFN-β is also used in cancer treatment (55). IFNα/β can nonetheless have detrimental immunosuppressive effects during chronic infection with certain viruses (56, 57). These contrasting roles of IFNα/β in controlling or exacerbating disease are a central feature of these pleiotropic cytokines (58). Similarly, IFNα/β might contribute to the development of autoimmune diseases such as lupus or psoriasis (59–61), whereas IFN-β can be beneficial in a large proportion of multiple sclerosis patients (62). In another setting, although IFNα/β are critical in the defense against bacteria (63–67), they could also impede antibacterial immunity by inducing apoptosis of immune cells, by suppressing inflammatory cytokine release, by responsiveness to IFN-γ, or by promoting IL-10 production (68–71).

In general terms, IFNα/β boosts pro-inflammatory cytokine/chemokine production and activates adaptive immunity, but also has diverse roles in immunity and, depending on context, might also suppress immune responses. IFN-α and IFN-β share a common receptor with apparently redundant functions. The two cytokines are used differently for treatment, and IFN-α is highly produced by plasmacytoid dendritic cells (47). The differences between IFN-α and IFN-β probably derive from the weak IFN-α binding to their common receptor (72).

### IFN-β Neutralization in Treating Hyperinflammation in Acute Sepsis

Several studies in IFN-β- and IFNAR-deficient mouse models of LPS- or TNF-α-induced septic shock suggest a pro-inflammatory role for IFN-β in septic shock (Table 1) (73–75). These observations support the idea of IFN-β or IFNAR neutralization as an adjunctive immunomodulatory therapy for sepsis (5). This view was corroborated by a subsequent report showing that IFNAR is needed for CLP-triggered sepsis development (76). An anti-IFNAR antibody also reduced sepsis symptoms and was functional even when injected after CLP induction, thus reinforcing the potential of IFN-β signaling inactivation as a therapeutic approach for sepsis (76). Sepsis improvement by the anti-IFNAR antibody precludes doubts about the relevance of data from mice in which IFN signaling is genetically abolished, based on the supposition that knockout mice might not reproduce the exact function of the eliminated gene. Studies in which septic shock was reduced by IFN-α (77) or IFN-β delivery (78) that suggest an anti-inflammatory effect for these cytokines need to be reinterpreted. Perhaps, the injected cytokine dose in these two studies elicits non-physiological effects that increase survival, as there is a striking difference between physiological IFN-β levels and those used for treatment (79). The role of IFN-β appears to lie more in propagating the inflammatory response than in initiating it, as its effect differs from that of TNF-α, a potent pro-inflammatory agent that causes septic shock in mice (75). In accordance with this view, TNF-α induces IFN-β production (58).

**Table 1:** **Role of IFN-β in modulating hyperinflammatory and immunosuppressive responses in mouse models and humans**.

	Models of IFN-β in sepsis	Reference
Hyperinflammation	IFN-β and IFN-α/β receptor (IFNAR) deficiency protect mice from LPS-induced septic shock	(73, 74)
	IFN-β and IFNAR deficiency protect mice from TNF-α-induced lethal shock	(75)
	IFNAR blockade protects mice in cecal ligation and puncture model	(76)
	IFN-α protects from LPS-induced lethality in mice	(77)
	IFN-β protects from LPS-induced septic shock in mice	(78)
	LPS treatment induces IFN-β expression in human monocytes	(31, 83)
	*Pseudomonas aeruginosa* infection induces IFN-β expression in human whole blood	(76)
Immunosuppression	IFN-β stimulation increases inflammatory response during endotoxin tolerance	(83)
	IFN-β is downregulated during endotoxin tolerance	(34, 83)
	IFNAR deficiency increases lethality in mouse model of delayed sepsis	(96)
	IFN-β is downregulated in immunosuppressed monocytes from sepsis patients	(83)

**Figure 1:**
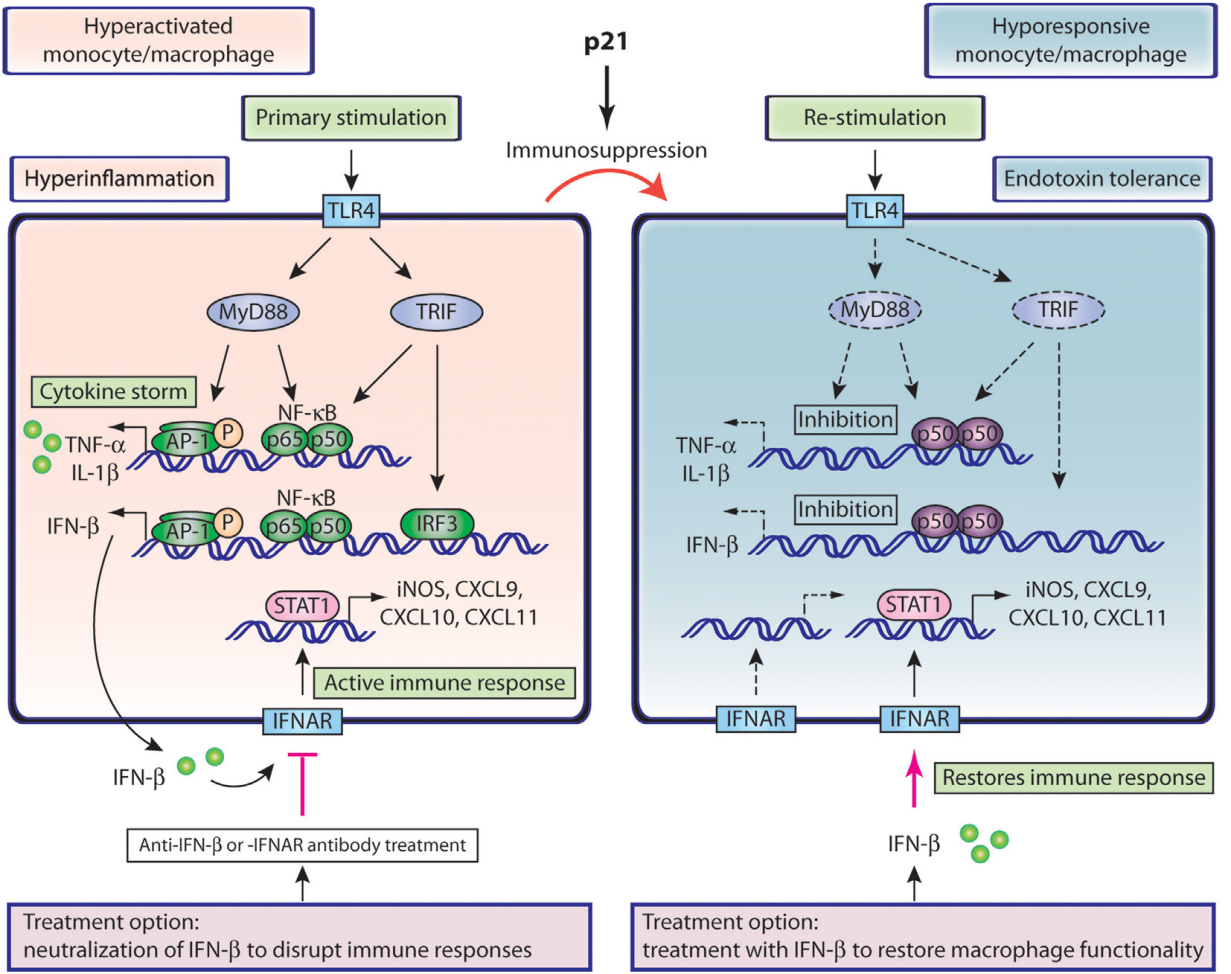
**Role of IFN-β in hyperactivated and hyporesponsive monocytes/macrophages**. Left, hyperactivated macrophages or monocytes present hyperinflammatory status and elevated IFN-β production. Secreted IFN-β interacts with its receptor and propagates the immune responses through iNOS and chemokine production. Neutralization of the IFN-β pathway interrupts these responses and could be beneficial in sepsis treatment. Right, hyporesponsive macrophages or monocytes associated with delayed sepsis or endotoxin tolerance arise from their hyperactivated counterparts, as a result of immunosuppression, driven by p21. TLR4 restimulation of such cells shows compromised activation pathways and inflammatory cytokine production, including IFN-β. IFN-β treatment could restore compromised monocyte functions and benefit immunosuppressed delayed sepsis patients.

TLR4 stimulation elicits hyperinflammation and IFN-β signaling in monocytes and macrophages as illustrated in Figure 1 (left). TLR4 triggering recruits MyD88 (myeloid differentiation primary-response protein 88) in order to activate both NF-κB and protein kinases (MAPK), which drives nuclear translocation of p65/p50 NF-κB and phospho-AP-1 and transcription of inflammatory cytokines. TLR4 stimulation also results in phosphorylation of IFN-regulatory factor 3 (IRF3), which in conjunction with NF-κB, induces expression of IFN-β (80, 81). Secretion of IFN-β activates the IFNAR complex in an autocrine manner. Subsequent STAT1 phosphorylation induces IFN-responsive elements such as iNOS, and chemokines such as CXCL9, CXCL10, and CXCL11 (Figure 1), which are potent white blood cell (WBC) attractors that further potentiate the immune response (31, 32, 82).

We recently showed that neutralization of IFNAR reduces iNOS and NO production as well as WBC-attracting chemokines in a mouse model of increased response to LPS (83). Neutralization of IFN-β signaling could thus reduce the propagation of inflammation and harmful physiological effects that depend on persistent NO production (83). These findings corroborate the idea that IFN-β does not act as an immediate hyperinflammatory factor, and its neutralization could be an attractive option that allows a greater margin for intervention, as the interval of hyperinflammation induction might be too short for effective treatment.

These data reinforce the idea that neutralizing IFN-β signaling could be a therapeutic option for acute sepsis patients. As suggested by Mahiou et al. (5), such an approach might be applied to relieve acute hyperinflammation, and caution should be taken to exclude patients with delayed sepsis to avoid aggravated immunosuppression.

### IFN-β in Restoring Functions of Compromised Immune Components

Boosting monocytes and other immune components to recover function is a prospective therapeutic approach for immunocompromised late sepsis patients. This idea is based on data showing that immunosuppressed monocytes from late sepsis patients recover HLA-DR levels and inflammation modulators after IFN-γ treatment (84). Although IFN-γ delivery to trauma or sepsis patients in clinical studies had some positive effects, it does not cure sepsis (7, 18). It is therefore suggested that a combination of IFN-γ treatment with GM-CSF, another monocyte booster, might prove more effective in treating immunosuppression in sepsis (7). The prevailing idea is that key cytokines or other pharmacological agents could revert immunosuppression of monocytes and other immune cells (37). Here we evaluate whether IFN-β could be included in the chart of promising factors to alleviate immunosuppression (7, 18).

IFN-β is essential for human monocyte inflammation (31), but is downmodulated in endotoxin-tolerized monocytes (34, 83). Importantly, IFN-β is also downregulated in immunosuppressed monocytes from sepsis patients (Table 1) (83), which implies that IFN-β downregulation could be critical for immunosuppression of monocytes in human sepsis, and that IFN-β treatment could reverse monocyte deactivation.

p21 was initially identified as a cell cycle and cyclin-dependent kinase 2 inhibitor (CDK2) (85). Other functions have been attributed to p21 (86, 87), and several studies designate it as an immune response modulator. p21 inhibits development of autoreactive T cells and autoimmunity (88–91). Moreover, it controls macrophage activation in septic shock and rheumatoid arthritis (92–94) and inhibits LPS-induced NF-κB activation, as well as endotoxin hyporesponsiveness of macrophages and monocytes (83). p21 regulates IFN-β levels in human monocytes, and monocytes from sepsis patients show high p21 levels, which correlate with low IFN-β expression.

These data suggest a model in which monocyte immunosuppression is controlled by p21, which promotes inhibitory p50–p50 over active p65–p50 NF-κB products (Figure 1, right). This p21 effect compromises production of inflammatory cytokines and IFN-β, and reduces iNOS induction and chemokine upregulation, which impairs WBC attraction and activation of innate and adaptive immunity (34, 47, 55, 83, 95). These IFNAR-dependent effects could theoretically be reestablished by an exogenous supply of IFN-β and thus restore monocyte functions and counteract immunosuppression (Figure 1, right). This model is further reinforced by our recent work showing that IFN-β treatment of endotoxin hyporesponsive macrophages increases expression of iNOS and CXCL11 (83). IFN-β can thus reestablish critical functional properties in immunosuppressed monocytes.

The role of IFN-β in controlling infection in long-term sepsis is supported by a mild CLP sepsis model. In such settings, WT mice survive the initial inflammatory shock, control bacteremia, and elude delayed death, whereas IFN-β-deficient mice, also unaffected by early inflammation, succumb to infection, and undergo late death (96). The data from this model, which in a way resembles delayed sepsis, show association of IFN-β expression with production of CXCL10, a chemokine that promotes homing of immune cells such as neutrophils (95, 96). In this mild CLP model, immunosuppression is possibly partial and IFN-β is produced. The results, however, support our view (Figure 1, right) that IFN-β, which must be supplied exogenously in severe immunosuppression, is essential for chemokine-mediated WBC attraction and antimicrobial action.

The model in Figure 1 (right) shows the possible effect of exogenous IFN-β in reversing monocyte hyporesponsiveness. This IFN-β effect can be extended to other immunosuppressed immune cells, as it can increase effector T cells, antibody responses in B cells, and activate innate immunity and antigen presentation (47, 95). As IFN-β can induce dendritic cell maturation (95), this cytokine could enhance the generation of dendritic cells, which are reduced by apoptosis in sepsis patients. Similarly to monocytes, exogenous IFN-β could reverse immunosuppressive aspects of dendritic cells, including IL-10 production, which inhibits IL-12 synthesis and T cell stimulation (45, 97).

Apart from its activating effect in monocytes and consistent with its pleiotropic antimicrobial responses, IFN-β could thus abrogate a wide range of sepsis-associated immunosuppressive responses. As little is known about its positive immunomodulatory effects in sepsis, establishing a role for IFN-β in antagonizing immunosuppression requires experimental evidence. Testing the effect of exogenous IFN-β in the “two hit” mouse model (28) could impart some early answers on its suitability for treatment of sepsis, which should be evaluated in human sepsis.

IFN-β has adverse immune effects that hinder microbial clearance in some systems (47, 98), such as proapoptotic effects on innate cells and T cells, inhibition of the IFN-γ pathway in macrophages, as well as generation of IL-10-producing Treg cells (58). The idea that IFN-β delivery might benefit immunosuppressed patients with delayed sepsis thus needs to be assessed meticulously. As the negative immune impact of IFN-β in infection is manifested in the context of certain but not all microbial infections, sepsis treatment might not be affected by these discordant IFN-β effects.

Much experimentation remains in order to elucidate the potential immunomodulatory effect of IFN-β in sepsis therapeutics and to estimate whether, in addition to IFN-β, any other stimulus could aid in efficient immune response reactivation in patients with delayed sepsis.

## Concluding Remarks

The failure of anti-TNF-α- or IL-1β-based therapies to decrease the death toll in sepsis patients has generated doubts as to whether cytokine-based treatments can be effective. Recent research has given new impetus to the field, and appropriate cytokine combinations are being considered for restoring suppressed immune functions in delayed sepsis patients. IFN-β-based therapeutic approaches such as neutralization could be used during the hyperinflammation phase of sepsis, but also during the immunosuppression phase, with IFN-β delivery to boost suppressed immunity. A thorough analysis of individual sepsis patients is needed before applying such radically opposed treatments for hyperinflammation or hyporesponsiveness. Patients with established immunosuppression might thus be a clearer target for IFN-β treatment to refurbish immune responses. Research is needed in mouse models and in humans to determine the precise mechanistic aspects and effectiveness of IFN-β-based treatments.

## Author Contributions

GR composed the figures and contributed in writing the manuscript; RS composed the final versions of the figures and organised the argument and the manuscript; MM and CM-A contributed to the intellectual content of the manuscript. DB conceived and wrote the manuscript; all authors read and approved the final version of the manuscript.

## Conflict of Interest Statement

The authors declare that the research was conducted in the absence of any commercial or financial relationships that could be construed as a potential conflict of interest.
